# Phytochemistry and Pharmacology of *Thymus broussonetii* Boiss

**DOI:** 10.1155/2021/6350035

**Published:** 2021-09-04

**Authors:** Hanae Naceiri Mrabti, Latifa Doudach, Naoual El Menyiy, Mohammed Bourhia, Ahmad Mohammad Salamatullah, Mohamed Reda Kachmar, Omar Belmehdi, Hamza El Moudden, Nidal Naceiri Mrabti, Hicham Harhar, Nasreddine El Omari, Abdelhakim Bouyahya

**Affiliations:** ^1^Laboratory of Pharmacology and Toxicology, Bio Pharmaceutical and Toxicological Analysis Research Team, Faculty of Medicine and Pharmacy, Mohammed V University in Rabat, BP 6203, Rabat, Morocco; ^2^Biomedical Engineering Department, National School of Arts and Crafts Rabat (ENSAM), Mohammed V University in Rabat, BP 6203, Rabat, Morocco; ^3^Faculty of Sciences Dhar El Mahraz, Sidi Mohamed Ben Abdellah University, Fez, Morocco; ^4^Laboratory of Chemistry-Biochemistry, Environment, Nutrition and Health, Faculty of Medicine and Pharmacy, Hassan II University, Casablanca 21100, Morocco; ^5^Department of Food Science & Nutrition, College of Food and Agricultural Sciences, King Saud University, P.O. Box 2460, Riyadh 11451, Saudi Arabia; ^6^Faculty of Sciences, Health and Environment Laboratory, Plant Protection Team, Moulay Ismail University, Meknes, BP 11201, Zitoun, Morocco; ^7^Biology and Health Laboratory, Department of Biology, Faculty of Science, Abdelmalek-Essaadi University, Tetouan, Morocco; ^8^Laboratory of Materials, Nanotechnology and Environment, Department of Chemistry, Faculty of Sciences, Mohammed V University in Rabat, Rabat, Morocco; ^9^Computer Chemistry and Modeling Team, Laboratory of Materieals, Modeling and Environmental Engineering, LIMME, Faculty of Sciences Dhar El Mehraz, Sidi Mohammed Ben Abdellah University, Fes, Morocco; ^10^Laboratory of Histology, Embryology, and Cytogenetic, Faculty of Medicine and Pharmacy, Mohammed V University, Rabat, Morocco; ^11^Laboratory of Human Pathologies Biology, Department of Biology, Faculty of Sciences, and Genomic Center of Human Pathologies, Faculty of Medicine and Pharmacy, Mohammed V University, Rabat, Morocco

## Abstract

*Thymus broussonetii* Boiss (*T. broussonetii*) is a rare medicinal and aromatic plant. It is widely used in traditional medicine to treat several diseases, including diarrhea, fever, cough, irritation, skin diseases, rheumatism, respiratory ailments, influenza, and digestion problems. In this review, we have critically summarized previous data on *T. broussonetii* about its phytochemistry, botanical and geographical distribution, toxicological investigation, and pharmacological properties. Using scientific research databases such as Wiley Online, SciFinder, ScienceDirect, PubMed, SpringerLink, Web of Science, Scopus Wiley Online, and Google Scholar, the data on *T. broussonetii* were collected and discussed. The presented data regrouped bioactive compounds and biological activities of *T. broussonetii*. The findings of this work showed that essential oils and extracts of *T. broussonetii* exhibited numerous pharmacological activities (*in vitro* and *in vivo*), particularly antibacterial, antifungal, antioxidant, anticancer, anti-inflammatory, insecticidal, antipyretic, antinociceptive, and immunological and behavioral effects. While toxicological studies of *T. broussonetii* essential oils and extracts are lacking, modern scientific tools revealed the presence of different classes of secondary metabolites such as terpenoids, alkaloids, flavonoids, tannins, coumarins, quinones, carotenoids, and steroids. *T. broussonetii* essential oils, especially from the aerial parts, exhibited potent antibacterial, antifungal, and antioxidant effects. An in-depth toxicological investigation is needed to validate the efficacy and safety of *T. broussonetii* extracts and essential oils and their secondary metabolites. However, further pharmacokinetic and pharmacodynamic studies should be performed to validate its bioavailability.

## 1. Introduction

*Thymus broussonetii* Boiss (*Thymus broussonetii*) belongs to the Lamiaceae family and the genus of *Thymus*. It is a small shrub of 40 cm in height and is endemic to Morocco, Algeria, and Tunisia [1]. It is known locally in Morocco as “Zaitra,” “Tazouknnit,” or “Azukni” [2, 3]. *T. broussonetii* is distributed on the Atlantic coast between 20 and 400 m altitude and is mainly located in arid and semiarid bioclimatic zones [4].

It is among the plants most used in Moroccan folk medicine against various illnesses such as urinary, nervous, genital, circulatory, skin, digestive, and respiratory diseases [2, 3]. It is also used to treat diabetes [3, 5, 6], cold, cough, fever, digestive disorders, and dolorous processes [7]. Other researchers have reported the use of this plant in food as a seasoning of traditional recipes (seasoning) and to flavor tea or milk [8]. Ethnobotanical surveys are the first step to identify the plant uses for each disorder. It provides information on the part used, the method of preparation, etc. However, the lack of plant information given by researchers in many surveys was repeatedly noticed. This is the case of several researchers who reported the use of *T. broussonetii* in folk medicine without mentioning the part used, the method of preparation, or/and the traditional use [9, 10].

Several classes of bioactive compounds, including flavonoids, alkaloids, terpenoids, tannins, coumarins, quinones, steroids, and carotenoids, have been identified in essential oils (EOs) and extracts of *T. broussonetii*, which explains its biological activities [11–25].

Using *in vitro* and *in vivo* pharmacological approaches, researchers reported the potential activity of *T. broussonetii* extracts and EOs. Essential oils from the aerial parts of *T. broussonetii* showed antibacterial effects against different pathogenic bacteria such as *Escherichia coli*, *Pseudomonas aeruginosa*, *Salmonella* sp., *Bacillus* sp., *Micrococcus luteus*, etc. Moreover, the antifungal effects of *T. broussonetii* EOs against numerous pathogenic fungi, including *Candida* sp., *Aspergillus brasiliensis*, and *Saccharomyces cerevisiae* were reported by Jamali et al. [20] and Smahane et al. [16, 20, 26]. *T. broussonetii* extracts and EOs exhibited antioxidant effects using well-known techniques such as DPPH and FRAP assays [11, 13, 20, 25, 27]. The anticancer properties of *T. broussonetii* EOs have also been reported against various tumor cell lines like P815 mastocytoma, CEM, and K-562 [12, 15, 21]. Moreover, *T. broussonetii* was revealed to exhibit anti-inflammatory activity [28], anticorrosive potential [23], insecticidal [19, 27, 29], antiparasitic [30], antipyretic [22], antinociceptive [31], immunological, and behavioral effects [31]. In addition, the acute toxicological investigations of *T. broussonetii* EOs have shown death cases and some signs of toxicity [22]. However, the mechanism of action by which the bioactive compounds of *T. broussonetii* extracts and EOs exhibited these pharmacological effects is lacking.

Due to the intensification of research on the pharmacological effects of *T. broussonetii* and its compounds in recent years, we have reviewed all studies on this plant; botanical description, geographical distribution, chemical composition, all pharmacological effects, and the prospects of *T. broussonetii*. To the best of our knowledge, this review is the first report providing a scientific database that highlighted several aspects related to *T. broussonetii* and suggested the future potential clinical applications of this plant.

## 2. Research Methodology *Thymus broussonetii* Boiss

In this work, data conacring botanical description, taxonomy, destruction, phytochemistry, and pharmacological activities of *T. broussonetii* were collected using different databases (Google Scholar, Web of Science, PubMed, Scopus, ScienceDirect, SpringerLink, SciFinder, and Wiley Online). The collected data were organized in several areas and highlighted. The chemical structures of T. *broussonetii* were drawn using ChemDraw Pro 8.0 software.

## 3. Results and Discussion

### 3.1. Botanical Description and Geographical Distribution

*Thymus broussonetii* is an evergreen plant that grows to a height of around 5 centimeters. Its flowers clustered toward the top of the stems in a dense ovate-cylindrical inflorescence with floral leaves broader than the leaves, often purple-colored, attenuate-sharp at the tip, ciliated at the margins and concealing the calyces, these 2-lipped, the upper shallowly toothed; pink corolla 2-3 times the length of the calyx, with a distinctly protruding narrow tube. It differs from subsp. hannonis (Maire) Morales by the subpetiolate leaves and bracts hairy only on the inner side [1]. *T. broussonetii* is an endemic plant to Morocco, Tunisia, and Algeria [1]. In Morocco, it is found in the Middle Atlantic, the High Atlas, and in the north of the kingdom [32].

### 3.2. Chemical Composition

**Figure 1:**
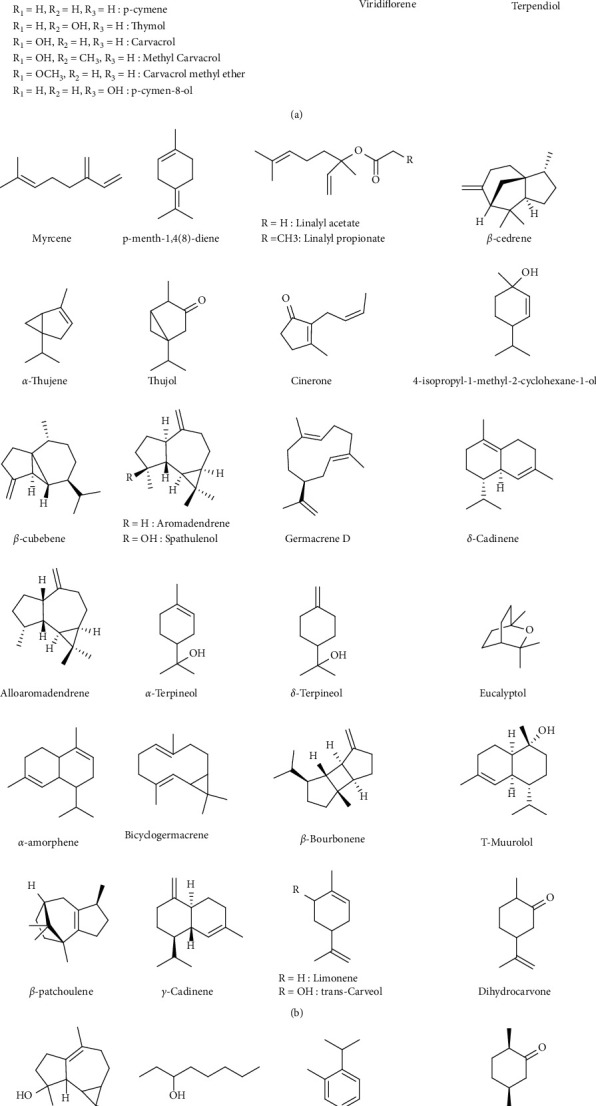
Chemical composition of T. *Thymus broussonetii*.

The secondary metabolites produced by *T. broussonetii* were the subject of numerous studies, almost all of which have been carried out on the aerial parts of this plant. The phytochemical screening of *T. broussonetii* extracts and EOs revealed its richness in phenolic compounds, in particular terpenoids, flavonoids, and phenolic acids. Analysis of *T. broussonetii* EOs by gas chromatography (GC) identified more than sixty terpenoids (Table 1; Figure 1).

The essential oil of *T. broussonetii* is mainly composed of spathulenol, eucalyptol, 1,8-cineole, *β*-caryophyllene, terpinolene, camphene, limonene, myrcene, sabinene, terpineol, terpinene, *p*-cymene, o-cymene, *α*-thujene, *α*-pinene, camphor, bornyl acetate, borneol, thymol, linalool, and carvacrol [11–24].

Chemical variability was observed in the composition of *T. broussonetii* extracted by different methods. Zerrifi et al. [17] have found that *T. broussonetii* EOs are rich in oxygenated monoterpenes (64.5%), monoterpene hydrocarbons (29.0%), sesquiterpene hydrocarbons (5.8%), and oxygenated sesquiterpenes (0.4%), while oxygenated sesquiterpenes had the lowest percentage. The carvacrol was the main compound [17].

The same results were found by Jamali et al. [20]. For the *T. broussonetii* essential oil from Essaouira (Morocco), it consisted mainly of oxygenated monoterpenes (64.5%), while the oxygenated sesquiterpenes were poorly represented (0.4%). The main component was carvacrol (43.4%), followed by thymol (12.3%) [20].

Carvacrol (39.51%) as the main constituent was also found by Chebli et al. [23]. The other components were o-cymene (14.80%), *γ*-terpinene (10.32%), *α*-pinene (9.7%), thymol (7.9%), and 4-terpineol (3.22%) [23].

In another study, camphor (46.17%) was found to be the major component followed by *α*-terpineol (7.69%), eucalyptol (5.76), germacrene *D* (5.21%), and borneol (4.42%) of *T. broussonnetii* essential oil in Tamri region (Western high Atlas), Morocco [14]. In addition, linalool, *γ*-terpinene, *cis*-sabinene hydrate, *β*-caryophyllene, *p*-Menth-1,4(8)-diene, caryophyllene oxide, and carvenoneare were the main compounds identified in the essential oil of *T. broussonnetii* aerial parts [21].

In comparison with wild-harvested and cultivated *T. broussonnetii*, chromatographic analysis of their essential oil revealed the presence of 19 compounds, namely *α*-pinene (5.0%), *p*-cymene (5.2%), borneol (8.5%), *γ*-terpinene (8.9%), thymol (12.3%), and carvacrol (43.4%) for wild-harvested plants in Morocco, whereas the oil obtained from cultivated plants was characterized by a higher content of *α*-pinene (6.5%), *p*-cymene (7.2%), and carvacrol (60.8%) [13].

The chemical analysis of polar fraction from *T. broussonnetii* leaf extracts indicated the presence of flavonoids, tannins, coumarins, terpenoids, quinones, steroids, and carotenoids in the various extracts (aqueous extract, alcohol extract, and petroleum extract). Alkaloid compounds were not detected in the methanolic extract of plant leaves. In addition, flavonoids, tannins, coumarins, terpenoids, quinones, steroids, and carotenoids were the main compounds identified in the *T. broussonnetii* stem extracts [25].

### 3.3. Pharmacological Properties

#### 3.3.1. Antibacterial Activity

Several studies have shown the antibacterial effectiveness of different essential oils from the aerial part of *Thymus broussonetii* [28, 39, 40, 29, 41].  Table 2 summarizes all the studies which evaluated this activity in *Thymus broussonetii*, including the plant part used, type of extract, the antibacterial test, the strains studied, and the key results. The literature screening indicated that scientists had investigated the effect of *Thymus broussonetii* against the most critical pathogenic agents belonging to Gram-negative and Gram-positive bacteria. Indeed, Lattaoui and Tantaoui-elaraki, [34] assessed the antibacterial activity of the essential oil of *T. broussonetii* aerial part against three bacteria (*Staphylococcus aureus*, *Escherichia coli*, and *Bacillus megaterium).* The result of this study showed that *T. broussonetii* essential oils inhibited the growth of all bacterial strains with MIC values of 1, 3, and 4% (v/v) against *S. aureus*, *E. coli*, and *B. megaterium*, respectively. Belaqziz et al. [33] reported the antibacterial activity of *T. broussonetti* leaf EOs using agar disc diffusion against two Gram-positive bacteria, including *S. aureus* and *Bacillus subtilis,* and four Gram-negative bacteria, namely *E. coli*, *Salmonella* sp, *Vibrio cholerae*, and *Pseudomonas aeruginosa.* The results showed that the essential oil exhibited promising antibacterial power against the strains tested; *Bacillus subtilis* (Ф = 33 ± 0.4 mm), *S. aureus* (Ф = 19 ± 0.8 mm), *Salmonella* sp. (Ф = 9 ± 0.9 mm), *Escherichia coli* (Ф = 21 ± 0.1 mm), *Vibrio cholerae* (Ф = 40 ± 0.4 mm) and *P. aeruginosa* (Ф = 9 ± 0.1 mm). In another study, El Bouzidi et al. [13] tested the antibacterial activity of essential oils obtained from both wild and cultivated *T. broussonetii* using agar disc diffusion and macrodilution methods against *Salmonella* sp. (CCMM B17), *E. coli* (CCMM B4), *E. coli* (ATCC 25922), *Bacillus cereus* (ATCC 14579), *Bacillus subtilis* (ATCC 9524), *Micrococcus luteus* (ATCC10240), *S. aureus* (CCMM B3), and the clinically isolated strain, *Enterobacter cloacae.* Both EOs obtained from *T. broussonetii* (wild and cultivated) exhibited inhibitory activity on all the selected microorganisms, with inhibitory zones ranging between 23.33 and 53.67 mm and MIC values varied from 0.12 to 1.82 mg/mL. In fact, *Micrococcus luteus* was the most sensitive strain with MIC values of 53.50 and 53.67 mg/mL for wild and cultivated *T. broussonetii,* respectively, followed by *B. subtilis*, *B. cereus*, and *S. aureus.* However, Smahane et al. [26] investigated the inhibitory effect of *T. broussonetii* aerial part EOs against *S. aureus*, *E. coli*, and *P. aeruginosa* using disk diffusion and broth microdilution methods. The results revealed that all microorganisms tested were inhibited by essential oils with inhibitory zones ranging between 8.67 and 42.67 mm and MIC values ranged between 0.2 and 20 *μ*g/mL.

Recently, Zerrifi and collaborators determined the *in vitro* antibacterial activity of *T. broussonetii* aerial part EOs using paper disk diffusion and microdilution methods against *Microcystis aeruginosa*. According to this study, the essential oils exhibited promising antibacterial power against the strain tested with an inhibitory zone of 90 mm, and MIC and MBC values of 0.047 and 0.095 mg/mL, respectively [17].

#### 3.3.2. Antifungal Activity

The antifungal activity of *T. broussonetii* EOs against many fungal strains was reported in several works [13, 16, 18, 20, 26, 34]. The previous publications on the antifungal activity that studied the essential oils from aerial parts of *T. broussonetii* by different methods are summarized in Table 3.

Saad et al. [18] determined the *in vitro* antifungal efficacy of the essential oil from the aerial part against *Candida albicans* using the agar diffusion and macrodilution broth methods. Consequently, the zones of inhibition and MIC value were 38.5 mm and 0.25 *μ*g/mL, respectively. Moreover, Jamali et al. [20] evaluated the EOs from aerial parts of the studied plant for their antifungal action against *Candida albicans*, *Candida krusei*, *Candida glabrata*, and *Candida parapsilosis* using agar disc diffusion and microdilution methods. The results revealed a strong antifungal activity against all the fungi tested with zones of inhibition ranging from 49.33 to 51.17 mm and MIC value of 0.45 mg/mL. Using the same methods and the same fungal strains, El Bouzidi et al. [13] investigated the antifungal activity of EOs obtained from wild and cultivated *T. broussonetii*. Therefore, these oils inhibited the growth of all fungal species with MIC values of 0.45 and 0.45 mg/mL for wild and cultivated *Thymus broussonetii*, respectively. In another study, the essential oil of *T. broussonetii* was tested against two fungal strains (*Candida albicans* and *Aspergillus brasiliensis*). The results revealed a strong antifungal inhibition against *Candida albicans* with zones of inhibition of 35.67 ± 0.33 mm [26].

#### 3.3.3. Antioxidant Activity

Different studies have evaluated the antioxidant activity of extracts and EOs from different parts of *T. broussonetii* using well-known techniques such as DPPH and FRAP assays [11, 13, 20, 25, 27] (Table 4). Indeed, Jamali et al. [20] investigated the antioxidant activity of the essential oils from aerial parts of *T. broussonetii*, and the results showed that the essential oil exhibited an interesting anti-DPPH (IC_50_ = 97.48 ± 2.24 *μ*g/mL) and a high reducing power (EC_50_ = 167.86 ± 1.46 *μ*g/ml) compared with the standard antioxidants, quercetin, and BHT with IC_50_ values of 1.07 ± 0.01 and4.21 ± 0.08 *μ*g/mL, respectively, for DPPH and with EC_50_ values of 2.29 ± 0.1 and 7.09 ± 0.1 *μ*g/mL, respectively, for FRAP. In another study, the wild and cultivated *T. broussonetii* EOs were tested for their antioxidant activity by DPPH and ferric ion reduction assays. The results showed an interesting antioxidant effect of the wild and cultivated *T. broussonetii* EOs with IC_50_ values of 132.23 ± 3.09 and 145.83 ± 3.47 *μ*g/mL, respectively, for DPPH and with EC_50_ values of 167.87 ± 1.46 and 169.355 ± 2.04 *μ*g/mL, respectively, for FRAP [13]. Moreover, Ouariachi et al. [11] demonstrated that the essential oils from *T. broussonetii* possessed high antioxidant activity using DPPH (IC_50_ = 90 *μ*g/mL). On the other hand, Ahlam et al. [25] reported the antioxidant activity of the aqueous and methanol extracts from leaves and stems of *T. broussonetii* using FRAP and DPPH methods. The results revealed that both extracts exhibited a good antioxidant activity with FRAP capacity values ranging between 0.105 ± 0.021 and 1.579 ± 0.014 mg/mL and anti-DPPH power with IC_50_ values ranging between 0.132 ± 0.034 and 7.665 ± 0.411 mg/mL. The highest activity was observed in methanol extract from stems with EC_50_ and IC_50_ values of 0.105 ± 0.021 and 0.132 ± 0.034 mg/mL, respectively. On the other hand, essential oil showed a DPPH-radical-scavenging activity with IC_50_ = 13.24 ± 0.06 mg/mL [27].

#### 3.3.4. Anticancer Activity

The anticancer properties of *T. broussonetii* have also been studied. Indeed, some investigations tested the efficiency of *T. broussonetii* essential oils on many cell lines [12, 15, 21] (Table 5). Ait M'Barek et al. [15] evaluated the antiproliferative effect of *T. broussonetii* EOs from stem and leaves on human ovarian adenocarcinoma IGR-OV1 parental cell line OV1/P. The results showed that the EOs tested inhibited the proliferation of this adenocarcinoma with an IC_50_ value of 0.40 ± 0.02 (%v/v).

Moreover, *Thymus broussonetii* EOs extracted from flowers and leaves have been tested by Jaafari et al. [21] on the P815 mastocytoma cell line using MTT assay. In this study, the essential oils exhibited an important dose-dependent cytotoxic effect against the P815 cell line (IC_50_ = 0.016%).

In another study, the authors evaluated the cytotoxic activity of essential oils from two chemotypes of *T. broussonetii* against five tumor cell lines, namely P-815 (murine mastocytoma), K-562 (human chronic myelogenous leukemia), CEM (acuteT lymphoblastoid leukemia), and MCF 7 (human breast adenocarcinoma) and its counterpart resistant to gemcitabine (MCF -7 gem) using MTT assay. Consequently, cell viability showed a cell proliferation inhibition by the tested products in a dose-dependent manner with IC_50_ values ranging between 3.1 and 17.5% (v/v). Additionally, cell cycle analysis detected cell cycle arrest at S and G0/G1 phases in cells. This considerable activity might be due to the high content of thymol and carvacrol known for their promising anticancer effects *via* numerous mechanisms of action such as angiogenesis, inhibition of cell migration, autophagy, apoptosis, and cell cycle arrest [35, 36].

#### 3.3.5. Anti-Inflammatory Activity

The antiedema effects of hexane, chloroform, and methanol extracts of *T. broussonettii* were evaluated on croton oil-induced ear edema in mice. The chloroform extract showed the highest activity, reducing the oedematous response by 47%, the ID_50_ value of the indomethacin used as the reference drug (286 g/cm^2^) is three times higher than that of the chloroform extract 93 g/cm^2^. The chloroform extract of *T. broussonettii* possesses an anti-inflammatory activity ascribable to its triterpenic acid content; in fact, ursolic and oleanolic acid justify the edema inhibition observed. Ursolic acid was more potent than oleanolic acid with ID_50_ values of 56 and 132 g/cm^2^ corresponding to 0.12 and 0.29 mol/cm^2^, respectively [28] (Table 6).

#### 3.3.6. Anticorrosive Potential

The essential oils of *T. broussonnetii* at different concentrations (ranging from 0.05 to 2 g/L) were tested against corrosion on C38 steel in 1 M medium, HCl, using electrochemical impedance spectroscopy (EIS), potentiodynamic polarization, and weight loss methods. The essential oil was found to be rich in bioactive substances, mainly carvacrol (39.51%) followed by benzene, 1-methyl-2-(1-methylethyl) (14.80%), gammaterpinene (10.32%), alpha-pinene (9.7%), thymol (7.9%), and 3-cyclohexen-1-ol, 4-methyl-1-(1-methylethyl) (3.22%). Using the EIS test, the essential oil (2 g/L) inhibited the corrosion of metals and alloys in acid solutions with a percentage of 82.35% of the inhibition efficiency. The polarization studies showed that *T. broussonnetii* EOs inhibit both anodic metal dissolution and cathodic hydrogen reduction reactions. At the highest inhibition concentration, the maximum inhibition efficiency observed indicates that many molecules were adsorbed on the metal surface. At 2 g/L, the best efficiency obtained in the presence of essential oil was 81.63%. It has been noted that the inhibition efficiency increases with increasing temperature. The highest efficiency was 90% and reached 328 K. The inhibitory mechanism was probably achieved by chemical adsorption (chemisorption) of TBS molecules on the surface of carbon steel and this indeed increases with rising temperature [23] (Table 6).

#### 3.3.7. Insecticidal Activities

The *T. broussonetti* EOs were investigated for their insecticidal activity, using the larvae test sensibility technique. The chemical analysis by GC-MS showed that the major compounds of *T. broussonetii* essential oil were p-cymene (21.0%), borneol (16.5%), *α*-pinene (11.8%), and thymol (11.3%). The EOs of this plant proved larvicidal effectiveness against the fourth instar larvae of *Culex pipiens* and were significantly higher at the dose of 0.125 ppm compared to the control. The lethal concentration 50 (LC_50_) during exposure of the insect population to EOs at 24 hours was 0.23, and the effective toxicity on *C. pipiens* larvae was associated with the thymol compound of thyme oil [19] (Table 6).

#### 3.3.8. Antipyretic Activity

At a dose of 200 mg/kg b.w., *T. broussonetii* aqueous, butanol, and ethyl acetate extracts were investigated *in vivo* for their antipyretic effect on yeast-induced fever. In normothermic rats, the extracts were tested to determine whether the antipyretic activity is related to a hypothermic effect. Indeed, all extracts significantly reduced rectal temperature in febrile animals. However, they did not induce hypothermia in normal rats. Besides, an inhibition of platelet aggregation has been observed by acting in the same way as NSAI drugs. Furthermore, extracts of *T. broussonetii* contain many types of compounds such as triterpenes, saponins, tannins, flavonoids, and several salicylates. The presence of these compounds can enhance this antipyretic activity [22] (Table 6).

#### 3.3.9. Antinociceptive

The immunostimulatory and neurotropic antistress effects of extracts (aqueous, ethyl acetate, and butanolic extracts) and EOs of *T. broussonetii* were evaluated at three doses. Therefore, the aqueous and ethyl acetate extracts showed the best results. In fact, thyme extracts increased the number of leucocyte categories studied, in particular polynuclear cells, total lymphocytes, TCD4+, TCD8+, and NK cells. It has been suggested that intraperitoneal administration of *T. broussonetii* extracts has a potent direct effect on leucocytes *in vivo*. In contrast, this assumes that the two extracts partially prevent stress-induced disturbances in the rate of leukocytes. The ethyl acetate extract inhibited the increase in polynuclear cells caused by stress, increased lymphocytes, and decreased polynuclear counts in the stressed mice treated with the aqueous extract compared to the stressed mice [31].

*T. broussonetii* was investigated to study the behavioral effects using the light/dark box test. At 12 mg/kg, the aqueous extract increased the number of transitions and the number of traversed squares and decreased the time spent in the dark compartment. The ethyl acetate extract increased both the number of traversed squares and the number of transitions without affecting the time spent in the dark compartment. The aqueous extract exerted an anxiolytic effect on the animals, while it could rather enhance locomotor and exploratory activities. The improvement in animal activity observed in the light/dark box after treatment with the aqueous extract is rather due to its anxiolytic-like effect and the ethyl acetate extract improved exploratory and locomotor activities in mice (Table 6).

#### 3.3.10. Antiparasitic Activity

In another work, the effect of *T. broussonetii* EOs was assessed on the experimental transmission of *Toxoplasma gondii* cysts in mice. These oils were administered orally (20 *μ*g/animal) at the infection time and thereafter for several days. In mice given the essential oils, no cyst was observed. In addition, no disorder was noted in the control animals given the thyme EOs [30] (Table 6).

#### 3.3.11. Insecticidal Activity

The insecticidal activity of *T. broussonetii* EO was screened using the contact toxicity assay. The oil proved insecticidal effectiveness against *Tribolium castaneum* Herbst. After 24 h of treatment, the LD_50_ and LD_90_ were 0.08 and 0.19 *μ*l/cm^2^, respectively. These results suggest that the contents of thyme EOs, in particular those obtained from the genus *Thymus*, have a good botanical bioinsecticide potential against *Tribolium castaneum* Herbst [29].

The insecticidal activity of the EO of this plant was examined against *Tribolium castanum* by the contact toxicity assay. The essential oil exhibited the highest insecticidal activity with a median lethal time (TL_50_) of 1.5 *μ*L/cm^2^ with LT_50_ (lethal time required to kill 50% of the exposed insects) values of 30,36 (24,62–38,48) at a dose of 1 *μ*l/cm^2^ and 4,81(3,8–5,99) at a dose of 1,5 *μ*l/cm^2^, respectively and a LT_90_ (lethal time required to kill 90% of the exposed insects) of 222,78(138,62–475,59) at a dose of 1 *μ*l/cm^2^ and 16,07 (11,4–30,08), respectively. The *Thymus broussonnetii* Boiss EO could act as a substitute for biopesticide and reduce the harmful impact of chemical insecticides on the environment and humans [27] (Table 6).

#### 3.3.12. Immunological and Behavioral Effects

The antinociceptive effect of aqueous, butanol, and ethyl acetate extracts of *T. broussonetii* was studied using thermal and chemical nociception models and naloxone (a nonselective opioid antagonist) to determine the role of the opioid system in the antinociceptive activity of these extracts. To determine the phytoconstituents of the extracts tested, phytochemical screening was carried out, which revealed the presence of tannins in all the extracts. Quinones, saponins, and flavonoids were detected in butanol and ethyl acetate extracts, while terpenes were only identified in the ethyl acetate extract [31].

The butanol and aqueous extracts showed an antinociceptive effect in both phases of formalin (50–300 mg/kg), tail immersion, and writing tests. At the same time, only the nociceptive response of the second phase was significantly reduced by the ethyl acetate extract (100–300 mg/kg). In the first and second phases, the aqueous extract was the most effective, with ED_50_ values of 177 (147–200) and 134 (95–170) mg/kg, respectively. The aqueous extract (200 mg/kg) showed a potent effect and significantly reduced the number of writhes induced by acetic acid, with 88.9% of writhes inhibition compared to those of ethyl acetate (69%) and butanol (63%) extracts. These obtained proved that *T. broussonetii* contains active compounds (polar and nonpolar) having antinociceptive activity with distinct mechanisms of action [31] (Table 6).

### 3.4. Toxicological Investigations

An acute toxicity screening was carried out for *T. broussonetii* EOs in order to verify their harmlessness to avoid a possible overdose and to properly determine the toxicological profile of the *T. broussonetii* species. This was assessed using the Leitchfield and Wilcoxon method, and the effective lethal dose (LD_50_) was measured. Subsequently, signs of toxicity such as diarrhea, convulsion, piloerection, motor coordination, and behavioral changes (excitation and twitches) were determined. For the groups receiving the dose of 1 g/kg, the change in body weight was also determined. On the other hand, thymol (36.7%) and borneol (21.9%) were the two major compounds, followed by *p*-cymene (7.6%) and *β*-pinene (0.7%). At a dose of 2 mg/kg, some cases of death and signs of toxicity were recorded. The LD_90_s and LD_50_s were estimated to be 7.31 (5.64–13.54) and 4.47 (3.6–6.72) g/kg, respectively [22].

## 4. Conclusion and Perspectives

Here, the phytochemistry, toxicology, and pharmacological properties of *T. broussonetii* were highlighted. Phytochemical studies of this species showed its richness in numerous bioactive compounds, exhibiting important biological effects. Pharmacological investigations confirmed the safety of this plant. However, these investigations must be further investigated using several toxicological reports at several different doses and time periods. Pharmacological biology explorations demonstrated that *T. broussonetii* essential oils and extracts exhibit important and remarkably antimicrobial, anticancer and, anti-inflammatory properties. These investigations were conducted using *in vitro* approaches, and therefore, further *in vivo* examinations should be performed to explore the pharmacological properties of T. broussonetii importantly. Moreover, mechanisms related to the biological effects of *T. broussonetii* and its bioactive compounds should also be explored to validate their pharmacodynamic actions.

## Figures and Tables

**Table 1:** Chemical composition of *T. broussonetii*.

Parts	Extracts/EOs	Compounds groups	Compounds	References
Leaves	Essential oil	Terpenoids	Borneol, *p*-cymene, carvacrol, camphene, *α* -terpinene, *α*-Pinene, trans-sabinene hydrate, caryophyllene oxide, (E)-*β*-caryophyllene, Bornyl acetate, carvacrol methyl ether, camphor, Linalool, cis-sabinene hydrate, 4-terpineol, p-cymen-8-ol, Thymol, trans-verbenol, 1-octen-3ol, 1,8-cineol, *β*-pinene	[11]
Flowers and leaves	Essential oil	Terpenoids	(E)-*β*-caryophyllene, *γ*-terpinene, *p*-cymene, carvacrol, thymol, 4-terpineol, *β*-pinene, terpendiol, borneol, caryophyllene oxide, geraniol formate, p-menth-1,4(8)-diene, linalyl propionate, *β*-cadrene, thujol, cinerone, 4-isopropy-lM-2-cyclohexane-1-ol, 1-octen-3-ol	[12]
Aerial parts	Essential oil	Terpenoids	*α*-pinene, *p*-cymene, carvacrol, viridiflorene, borneol, *γ*-terpinene, myrcene, camphene, *α*–thujene, aromadendrene, caryophyllene oxide, *α*–terpinene, *β*–pinene, thymol, germacrene *D*, *δ*-cadinene, linalool	[13]
Aerial parts	Essential oil	Terpenoids	Camphor, *α*–terpineol, eucalyptol, germacrene *D*, borneol, terpinen-4-ol, bicyclogermacrene, *β*–caryophyllene, *β*-bourbonene, spathulenol, *δ*–terpineol, bornyl acetate, caryophyllene oxide, T-muurolol, *γ*–cadinene, thymol, trans-sabinene hydrate, linalool, cis-sabinne-hydrate, limonene, *p*-cymene, dihydrocarvone, trans-carveol, *δ*–cadinene, alloaromadendrene, carvacrol	[14]
Leaves	Essential oil	Terpenoids	Carvacrol, p-cymene,*γ* -terpinene, thymol, *β*--pinene, 4-terpineol, borneol, linalyl propionate, p-menth-1,4(8)-diene, geraniol formate, cinerone, carvacrol methyl ether, 4-isopropyl-1M-2cyclohexane-1-ol,1-octen-3-ol	[15]
	Essential oil	Terpenoids	Carvacrol, p-cymene, *α*-pinene, *α*-terpinene, 3-octanol, myrcene, *α*-terpineol, borneol, linalyl acetate, linalool, *β*--pinene, methyl carvacrol, p-cymen-8-ol, p-mentha-1,4(8)-diene, limonene, camphene, *γ*-terpinene, thymol	[16]
Aerial parts	Essential oil	Terpenoids	Carvacrol, thymol, *γ*-terpinene, borneol, *p*-cymene, *α*-pinene, camphene, myrcene, *α*-terpinene, *α*-thujene, limonene, *β*-pinene, linalool	[17]
Aerial parts	Essential oil	Terpenoids	Thymol, carvacrol, borneol, viridiflorene, spathulenol, aromadendrene, camphene, *α*-terpineol, O-cymene, terpinene-4-ol, *γ*-terpinene, alloaromadendrene, *γ*-cadinene, *α*-pinene, *cis*-dihydrocarvone, trans-sabinene hydrate, *α*-amorphene, *β*-patchoulene, *β*-cubebene, isospathulenol	[18]
Leaves	Essential oil	Terpenoids	*p*-cymene, borneol, *α*-pinene, thymol, camphene, *γ*-terpinene, carvacrol, Ledene, Limonene, Myrcene, Aromadendrene, *β*-pinene, *α*-thujene,*α*-terpinene, terpinen-4-ol, dihydrocarvone, allo-Aromadendrene, *β*-caryophyllene, *cis*-sabinene hydrate, tricyclene, sabinene, *α*-phellandrene, *p*-Mentha-1,4(8)-diene, linalool, *γ*-muurolene, spathulenol	[19]
Aerial parts	Essential oil	Terpenoids	Carvacrol, thymol, borneol, *γ*-terpinene, *p*-cymene, camphene, *α*-pinene, myrcene, viridiflorene, *α*-terpinene, *α*-thujene, aromadendrene, *β*-pinene, limonene, caryophyllene oxide, tricyclene, *δ*-cadinene, alloaromadendrene, germacrene *D*, linalool, and limonene	[20]
Aerial parts	Essential oil	Terpenoids	Borneol, thymol, *p*-cymene, *γ*-terpinene, carvacrol, 4-terpineol, linalyl propionate, camphor, *δ*-3-carene, camphene, *β*-pinene, geraniol formate, p-menth-1,4(8)-diene, p-mentha-1,8-diene, 4-isopropyl-1M-2 cyclohexane-1-ol, terpinene-1-ol, carvenone, bornyl acetate, cinerone, alloaromadendrene, (E)-*β*-caryophyllene, *α*-muurolene, *β*-cedrene, *α*-cadinene, caryophyllene oxide, germacrene D	[21]
	Essential oil	Terpenoids	Thymol, borneol, carvacrol, *p*-cymene, *δ*- terpinene, camphene, spathulenol, myrcene, *α*-terpineol, aromadendrene, limonene, *β*--pinene, *α*-terpinene	[22]
Aerial parts	Essential oil	Terpenoids	Carvacrol, o-cymene, *γ*.-terpinene, *α*-pinene, thymol, (+)-4-carene, 4-terpineol, *α*-thujene, tau-cadinol, spathulenol, limonene, *β*-caryophyllene, and camphene	[23]
Aerial parts	Essential oil	Terpenoids	Thymol, *α*-pinene, *β*-caryophyllene, carvacrol, *γ*-terpinene, borneol	[24]
Leaves	Methanolic extract	Alkaloids	−	[25]
	Alcohol extract	Flavonoids	+	
	Aqueous extract	Tannins	+	
	Ethanolic extract	Coumarins	+	
	Methanolic extract	Terpenoids	+	
	Petroleum extract	Quinones	+	
		Steroids	+	
	Aqueous extract	Carotenoids	+	
Stems	Methanolic extract	Alkaloids	−	
	Alcohol extract	Flavonoids	+	
	Aqueous extract	Tannins	+	
	Ethanolic extract	Coumarins	+	
	Methanolic extract	Terpenoids	+	
	Petroleum extract	Quinones	+	
		Steroids	+	
	Aqueous extract	Carotenoids	+	

**Table 2:** Antibacterial effects of *T. broussonetii*.

Used parts	Extracts	Used methods	Tested strains	Key results	References
Aerial part	Essential oil	Agar disk diffusion method Broth microdilution method	*Staphylococcus aureus*	Ф = 42.67 ± 1.45 mm	[26]
				MIC = 0.2 *μ*l/mL	
				MBC = 0.6 *μ*l/mL	
			*Escherichia coli*	Ф = 29.33 ± 0.54 mm	
				MIC = 1.3 *μ*l/mL	
				MBC = 1.3 *μ*l/mL	
			*Pseudomonas aeruginosa*	Ф = 8.67 ± 1.20 mm	
				MIC = 20 *μ*l/mL	
				MBC ≥ 80 *μ*l/mL	

Aerial part	Essential oil	Agar disc diffusion Broth macrodilution method	*Staphylococcus aureus*	Ф = 35.00 ± 1.00 mm	[13]
				MIC = 0.9 mg/mL	
				MMC = 0.9 mg/mL	
			*Bacillus subtilis*	Ф = 49.67 ± 1.53 mm	
				MIC = 0.23 mg/mL	
				MMC = 0.23 mg/mL	
			*Bacillus cereus*	Ф = 48.67 ± 1.15 mm	
				MIC = 0.23 mg/mL	
				MMC = 0.23 mg/mL	
			*Micrococcus luteus*	Ф = 53.50 ± 1.00 mm	
				MIC = 0.12 mg/mL	
				MMC = 0.12 mg/mL	
			*Escherichia coli* 1 ATCC 25922	Ф = 30.17 ± 1.00 mm	
				MIC = 0.90 mg/mL	
				MMC = 0.90 mg/mL	
			*E. coli* 2 CCMM B4	Ф = 29.67 ± 1.53 mm	
				MIC = 0.90 mg/mL	
				MMC = 0.90 mg/mL	
			*Enterobacter cloacae*	Ф = 27.33 ± 0.58 mm	
				MIC = 0.90 mg/mL	
				MMC = 0.90 mg/mL	
			*Salmonella* sp.	Ф = 31.67 ± 1.53 mm	
				MIC = 0.90 mg/mL	
				MMC = 0.90 mg/mL	
			*Staphylococcus aureus*	Ф = 34.83 ± 1.04 mm	
				MIC = 0.91 mg/mL	
				MMC = 0.91 mg/mL	
			*Bacillus subtilis*	Ф = 49.00 ± 1.00 mm	
				MIC = 0.23 mg/mL	
				MMC = 0.23 mg/mL	
			*Bacillus cereus*	Ф = 47.33 ± 1.15 mm	
				MIC = 0.23 mg/mL	
				MMC = 0.23 mg/mL	
			*Micrococcus luteus*	Ф = 53.67 ± 1.15 mm	
				MIC = 0.12 mg/mL	
				MMC = 0.12 mg/mL	
			*Escherichia coli* 1 ATCC 25922	Ф = 27.5 ± 1.53 mm	
				MIC = 0.91 mg/mL	
				MMC = 0.91 mg/mL	
			*E. coli* 2 CCMM B4	Ф = 29.33 ± 1.53 mm	
				MIC = 0.91 mg/mL	
				MMC = 0.91 mg/mL	
			*Enterobacter cloacae*	Ф = 23.33 ± 1.53 mm	
				MIC = 1.82 mg/mL	
				MMC = 1.82 mg/mL	
			*Salmonella* sp.	Ф = 31.33 ± 1.53 mm	
				MIC = 0.91 mg/mL	
				MMC = 0.91 mg/mL	

Aerial part	Essential oil	Agar diffusion method	*Bacillus subtilis*	Ф = 33 ± 0.4 mm	[33]
			*Staphylococcus aureus*	Ф = 19 ± 0.8 mm	
			*Salmonella* sp.	Ф = 19 ± 0.9 mm	
			*Escherichia coli*	Ф = 21 ± 0.1 mm	
			*Vibrio cholerae*	Ф = 40 ± 0.4 mm	
			*Pseudomonas aeruginosa*	Ф = 9 ± 0.1 mm	

Aerial part	Essential oil	Disc diffusion method	*Microcystis aeruginosa*	Ф = 90 ± 0.00 mm	[17]
				MIC = 0.047 mg/mL	
				MBC = 0.095 mg/ml	
Aerial part	Essential oil	Agar diffusion method	*Staphylococcus aureus*	No measurable zone of inhibition	[34]

Aerial part	Essential oil	Agar diffusion method	*Escherichia coli*	No measurable zone of inhibition	[16]
				MIC = 1%	
			*Staphylococcus aureus*	MIC = 3%	
			*Bacillus megaterium*	MIC = 4%	

**Table 3:** Antifungal activity of *T. broussonetii*.

Used parts	Extracts	Used methods	Tested strains	Key results	References
Aerial parts	Essential oil	Agar disc diffusion Broth microdilution method	*Candida albicans*	Ф = 50.00 ± 1.00 mm	[20]
				MIC = 0.45 mg/mL	
				MMC = 0.45 mg/mL	
			*Candida krusei*	Ф = 49.67 ± 1.53 mm	
				MIC = 0.45 mg/mL	
				MMC = 0.45 mg/mL	
			*Candida glabrata*	Ф = 49.33 ± 1.53 mm	
				MIC = 0.45 mg/mL	
				MMC = 0.45 mg/mL	
			*Candida parapsilosis*	Ф = 51.17 ± 0.76 mm	
				MIC = 0.45 mg/mL	
				MMC = 0.45 mg/mL	
	Essential oil	Agar disc diffusion Broth microdilution method	*Candida albicans*	Ф = 50.00 ± 1.00 mm	[13]
				MIC = 0.45 mg/mL	
				MMC = 0.45 mg/mL	
			*Candida krusei*	Ф = 49.67 ± 1.53 mm	
				MIC = 0.45 mg/mL	
				MMC = 0.45 mg/mL	
			*Candida glabrata*	Ф = 49.33 ± 1.53 mm	
				MIC = 0.45 mg/mL	
				MMC = 0.45 mg/mL	
			*Candida parapsilosis*	Ф = 51.17 ± 0.76 mm	
				MIC = 0.45 mg/mL	
				MMC = 0.45 mg/mL	
			*Candida albicans*	Ф = 49.67 ± 1.15 mm	
				MIC = 0.46 mg/mL	
				MMC = 0.46 mg/mL	
			*Candida krusei*	Ф = 47.33 ± 1.53 mm	
				MIC = 0.46 mg/mL	
				MMC = 0.46 mg/mL	
			*Candida glabrata*	Ф = 48.50 ± 0.50 mm	
				MIC = 0.46 mg/mL	
				MMC = 0.46 mg/mL	
			*Candida parapsilosis*	Ф = 50.00 ± 1.00 mm	
				MIC = 0.46 mg/mL	
				MMC = 0.46 mg/mL	

Aerial parts	Essential oil	Agar diffusion method macrodilution broth method	*Candida albicans*	Ф = 38.5 ± 0.70 mm	[18]
				MIC = 0.25 *μ*g/mL	

Aerial parts	Essential oil	Agar diffusion method	*Candida albicans*	Slightly more sensitive in presence of 0.2% oil	[34]
			*Aspergillus niger*	Lower sensitivity relatively resistant	

Aerial parts	Essential oil	Agar diffusion method	*Saccharomyces cerevisiae*	MIC = 3%	[16]
			*Candida albicans*	MIC = 3%	
			*Zygorrhynchussp*	MIC = 4%	
			*Aspergillus niger*	MIC = 3%	

Aerial part	Essential oil	Agar disk diffusion method Broth microdilution method	*Candida albicans*	Ф = 35.67 ± 0.33 mm	[26]
				MIC = ND	
				MBC = ND	
			*Aspergillus brasiliensis*	Ф = ND	
				MIC = ND	
				MBC = ND	

**Table 4:** Antioxidant effects of *T. broussonetii*.

Used parts	Extracts	Used methods	Key results	References
Leaves	Aqueous extract	DPPH	IC_50_ = 22.61 ± 1.022 mg/mL	[25]
	Methanol extract		IC_50_ = 6.484 ± 0.190 mg/mL	
Stems	Aqueous extract		IC_50_ = 7.665 ± 0.411 mg/mL	
	Methanol extract		IC_50_ = 0.132 ± 0.034 mg/mL	
Leaves	Aqueous extract	FRAP	EC_50_ = 0.597 ± 0.013 mg/mL	
	Methanol extract		EC_50_ = 1.579 ± 0.014 mg/mL	
Stems	Aqueous extract		EC_50_ = 0.489 ± 0.011 mg/mL	
	Methanol extract		EC_50_ = 0.105 ± 0.021 mg/mL	
Aerial parts	Essential oil	DPPH	IC_50_ = 13.24 ± 0.06 mg/mL	[27]
Aerial parts	Essential oil	DPPH	IC_50_ = 90 *μ*g/mL	[11]
Aerial parts (wild)	Essential oil	DPPH	IC_50_ = 132.23 ± 3.09 *μ*g/mL	[13]
		FRAP	EC_50_ = 167.87 ± 1.46 *μ*g/mL	
Aerial parts (cultivated)	Essential oil	DPPH	IC_50_ = 145.83 ± 3.47 *μ*g/mL	
		FRAP	EC_50_ = 169.355 ± 2.04 *μ*g/mL	
Aerial parts (wild)	Essential oil	DPPH	IC_50_ = 97.48 ± 2.24 *μ*g/mL	[20]
		FRAP	EC_50_ = 167.86 ± 1.46 *μ*g/mL	

**Table 5:** Anticancer effects of *T. broussonetii*.

Parts used	Extracts	Used methods	Cell lines	Key results	References
Leaves and stems	Essential oils	Crystal violet assay	The parental human ovarian adenocarcinoma cell line IGR-OV1 (OV1/P)	IC_50_ = 0.40 ± 0.02%(v/v)	[15]
Flowers and leaves	Essential oils (variety: TbA)	MTT assay	P815 mastocytoma cell line	IC_50_ = 4.7%(v/v)	[12]
			CEM	IC_50_ = 3.6%(v/v)	
			K-562	IC_50_ = 10%(v/v)	
			MCF -7	IC_50_ = 10%(v/v)	
			MCF -7 gem	IC_50_ = 8.9%(v/v)	
	Essential oils (variety: TbB)	MTT assay	P815 mastocytoma cell line	IC_50_ = 8.5%(v/v)	
			CEM	IC_50_ = 3.1%(v/v)	
			K-562	IC_50_ = 13.5%(v/v)	
			MCF -7	IC_50_ = 15.4%(v/v)	
			MCF -7 gem	IC_50_ = 17.5%(v/v)	
Flowers and leaves	Essential oils	MTT assay	P815 mastocytoma cell line	IC_50_ = 0.016%(v/v)	[21]

**Table 6:** Other pharmacological activities of *T. broussonetii*.

Activities	Used parts	Extracts	Experimental approaches	Key results	References
Anti-inflammatory activity	Leaves	n-hexane	Croton oil ear test in mice inhibition of the croton oil-induced ear edema in mice	Edema reduction = 9%	[28]
		Chloroform		Edema reduction = 47%	
		Chloroform + methanol		Edema reduction = 16%	
		Methanol		Edema reduction = -5%	
Anticorrosive activity	Aerial parts	Essential oils	Loss measurements and electrochemical techniques	82.35% inhibition efficiency at a dose of 2 g/L	[23]
Insecticidal activity	Aerial parts	Essential oils	Fourth instar larvae of *Culex pipiens*	LC_50_ = 0.23	[19]
Antiparasitic activity	Aerial parts	Essential oils	Oral administration (20 g/animal) at the time of infection and thereafter for several days	Absence of intracerebral cysts No anomalies	[30]
Antipyretic activity	Stem	Water, butanol, and ethyl acetate	Yeast-induced fever in rats	Significantly reduced the temperature in febrile rats	[37]
Acute toxicity	Aerial parts	Essential oils	Swiss mice (25–35 g)	LD_50_ = 2.66 g/kg	[22]
Antinociceptive activity	Leaves and stem	Water	Chemical and thermal models (*in vivo*)	Writhing inhibition = 88.9%	[31]
		Ethyl acetate and butanol		Writhing inhibition = 69%	
				Writhing inhibition = 62.8%	
Insecticidal activity	Aerial parts	Essential oils	Effect against adults of *Tribolium castaneum* herbst	LD_50_ = 0.08 *μ*l/cm^2^ LD_90_ = 0.19 *μ*l/cm^2^	[29]
Insecticidal activity	Aerial parts	Essential oils	Effect against *Tribolium castanum* pest foodstuffs	TL_50_ = 1.5 *μ*l/cm^2^	[27]
Immunological and behavioral activities	Leaves and stem	Water, butanol, and ethyl acetate	Tested the neurostimulant effects of the extracts	Increased (*in vivo*) the number of leukocyte categories studied	[31]

## Data Availability

The data used to support the findings of this study are included within the article.
